# CropGene: a software package for the analysis of genomic and transcriptomic data of agricultural plants

**DOI:** 10.18699/vjgb-25-35

**Published:** 2025-04

**Authors:** A.Yu. Pronozin, D.I. Karetnikov, N.A. Shmakov, M.E. Bocharnikova, S.D. Afonnikova, D.A. Afonnikov, N.A. Kolchanov

**Affiliations:** Institute of Cytology and Genetics of the Siberian Branch of the Russian Academy of Sciences, Novosibirsk, Russia Kurchatov Genomic Center of ICG SB RAS, Novosibirsk, Russia; Institute of Cytology and Genetics of the Siberian Branch of the Russian Academy of Sciences, Novosibirsk, Russia Kurchatov Genomic Center of ICG SB RAS, Novosibirsk, Russia; Institute of Cytology and Genetics of the Siberian Branch of the Russian Academy of Sciences, Novosibirsk, Russia Kurchatov Genomic Center of ICG SB RAS, Novosibirsk, Russia; Institute of Cytology and Genetics of the Siberian Branch of the Russian Academy of Sciences, Novosibirsk, Russia Kurchatov Genomic Center of ICG SB RAS, Novosibirsk, Russia; Institute of Cytology and Genetics of the Siberian Branch of the Russian Academy of Sciences, Novosibirsk, Russia Kurchatov Genomic Center of ICG SB RAS, Novosibirsk, Russia; Institute of Cytology and Genetics of the Siberian Branch of the Russian Academy of Sciences, Novosibirsk, Russia Kurchatov Genomic Center of ICG SB RAS, Novosibirsk, Russia; Institute of Cytology and Genetics of the Siberian Branch of the Russian Academy of Sciences, Novosibirsk, Russia Kurchatov Genomic Center of ICG SB RAS, Novosibirsk, Russia

**Keywords:** bioinformatics pipeline, software package, SNP, analyzing polymorphisms, identification of genes, биоинформатический конвейер, программный пакет, SNP, анализ полиморфизмов, идентификация генов

## Abstract

Currently, the breeding of agricultural plants is increasingly based on the use of molecular biological data on genetic sequences, which makes it possible to significantly accelerate the breeding process, create new plant varieties through genomic editing. These data have a large volume, variety and require a large amount of resources, both labor and computing, to analyze the costs. Data analysis of such volume and complexity can be effective only when using modern bioinformatics methods, which include algorithms for identifying genes, predicting their function, and evaluating the effect of mutation on plant phenotype. Such an analysis has recently become impossible without the use of integrated software systems that solve problems of different levels by executing computational pipelines. The paper describes the CropGene software package developed for the comprehensive analysis of genomic and transcriptomic data of agricultural plants. CropGene includes several blocks of bioinformatic analysis, such as analysis of gene variations, assembly of genomes and transcriptomes, as well as annotation of genes and proteins. CropGene implements new methods for analyzing long non-coding RNAs, protein domains, searching and analyzing polymorphisms, and genome-wide association research. CropGene has a user-friendly interface and supports working with various types of data, which greatly simplifies its use for researchers who do not have deep knowledge in the field of bioinformatics. The paper provides examples of the use of CropGene for the analysis of agricultural organisms such as Solanum tuberosum and Zea mays. With CropGene, genetic markers have been identified that explain up to 50 % of the variability in seed color parameters; potential genes that may become promising material for producing potato varieties; more than 100 thousand new long non-coding RNAs. Orthogroups were also found, the domain structure of which shows a marked similarity with the domain architecture of characteristic secreted A2 phospholipases. Thus, CropGene is an important tool for scientists and practitioners working in the field of agrobiotechnology and plant genetics.

## Abstract (ru)

В настоящее время селекция сельскохозяйственных растений все больше опирается на использование молекулярно-биологических данных о генетических последовательностях, что позволяет существенно ускорить селекционный процесс создания новых сортов растений за счет геномного редактирования. Эти данные имеют большой объем, разнообразны и требуют для анализа затрат большого количества ресурсов, как трудовых, так и вычислительных. Анализ данных с такими объемом и сложностью может быть эффективным лишь с применением современных методов биоинформатики, включающих алгоритмы идентификации генов, предсказания их функции, оценку влияния эффекта мутации на фенотип растений. Такой анализ в последнее время стал невозможным без использования интегрированных программных комплексов, решающих задачи разного уровня за счет выполнения вычислительных конвейеров. В статье описан программный комплекс CropGene, разработанный для комплексного анализа геномных и транскриптомных данных сельскохозяйственных растений. Система включает в себя несколько блоков биоинформатического анализа, таких как анализ вариаций генов, сборка геномов и транскриптомов, а также аннотация генов и белков. В комплексе реализованы новые методы анализа длинных некодирующих РНК, белковых доменов, поиска и анализа полиморфизмов и полногеномного исследования ассоциаций. В работе представлены примеры применения CropGene для анализа сельскохозяйственных организмов, таких как Solanum tuberosum, Zea mays. С помощью данного программного пакета найдены: генетические маркеры, объясняющие до 50 % изменчивости параметров окраски семян; потенциальные гены, которые могут стать перспективным материалом для получения сортов картофеля; более 100 тыс. новых длинных некодирующих РНК. Также обнаружены ортогруппы, доменная структура которых проявляет заметное сходство с доменной архитектурой характерных секретируемых фосфолипаз А2. Таким образом, CropGene представляет собой важный инструмент для ученых и практиков, работающих в области агробиотехнологий и генетики растений.

## Introduction

Key terms Intronic lncRNAs – overlap with the intron of a gene Antisense lncRNAs – oriented against the direction of transcription of a protein-coding gene Intergenic lncRNAs – located between two gene loci Genome-Wide Association Studies (GWAS) – a method of genome research aimed at finding statistical relationships between genetic variations and certain phenotype traits Transcriptome – a set of all transcripts present in a cell at a certain stage of development or under certain physiological conditions Gene network – a group of coordinated functioning genes interacting with each other both through their primary products (RNA and proteins) and through a variety of metabolites and other secondary products of gene network functioning

In contemporary agricultural science, the development of plant breeding strategies is increasingly dependent on the utilization of molecular biological data, particularly genetic sequence information. Genetic sequence information facilitates a significant acceleration of the breeding process (Khlestkina, 2014) and enables the creation of novel plant varieties through advanced genomic editing techniques. The extensive size, high dimension, and inherent complexity of these data sets demand substantial computational and labor resources for thorough investigation. The effective interpretation of such large-scale and intricate data is achievable only through the application of modern bioinformatics methodologies, which encompass algorithms for gene identification, functional annotation, and the assessment of mutational impacts on phenotypic expression. In recent years, the integration of computational modeling and deep learning algorithms has become indispensable for such analyses. Furthermore, the development of automated computational pipeline technologies is advancing to streamline and optimize data processing workflows within the field of bioinformatics.

The investigation of genetic and transcriptomic information in plant species involves numerous crucial endeavors, notably the examination of genetic variety. Genetic diversity is an important basis for identifying genes associated with resistance to biotic and abiotic stresses, as well as for developing novel, highly adaptive, and high-yielding crop varieties. The assessment of genetic diversity is conducted through a variety of genetic analysis methodologies. Notably, genetic markers play a pivotal role in such studies (Khlestkina, 2014). Among these markers, single-nucleotide polymorphisms (SNPs), which represent single-nucleotide substitutions occurring at varying frequencies within plant populations, are of particular significance (Sukhareva, Kuluev, 2018). SNP analysis is extensively employed to examine allelic polymorphism, analyze haplotypes and pedigrees, and facilitate genotyping and the construction of genetic maps.

In addition to SNP analysis, copy number variation (CNV) is employed to investigate genetic diversity. CNV represents a form of genetic polymorphism characterized by differences in the number of copies of specific genomic regions among individuals. These variations encompass deletions or duplications of individual genes or clusters of linked genes. CNVs can span extensive genomic regions, ranging from several kilobases to millions of base pairs, and play a significant role in contributing to genomic variability and phenotypic diversity.

Genome-wide information on SNPs across hundreds of samples can be obtained through next-generation highthroughput sequencing technologies. SNP identification is achievable using two primary strategies: whole-genome sequencing (WGS) and genotyping by sequencing (GBS) (Scheben et al., 2017). The GBS approach is notably faster and more cost-effective compared to WGS. This efficiency is achieved by sequencing genomic DNA fragments only in proximity to restriction enzyme recognition sites, thereby reducing the overall sequencing cost. However, this method results in fragmented genome coverage and yields a lower density of SNPs compared to comprehensive whole-genome sequencing. Despite these limitations, the data generated through GBS are sufficiently robust to characterize the genetic diversity of agricultural plant populations with acceptable accuracy. Furthermore, GBS data are widely utilized in genome-wide association studies (GWAS), a powerful tool for identifying genes associated with complex quantitative traits (Burghardt et al., 2017).

In addition to providing fundamental insights into the genetic mechanisms underlying traits of interest, GWAS also facilitate the discovery of genetic markers that can be directly applied to breeding programs (Tsai et al., 2010; Zatybekov et al., 2017; Larkin et al., 2019; Muqaddasi et al., 2020).

Another area of bioinformatics research in agricultural plants involves the assembly of genomes and transcriptomes. Genome assembly represents a foundational step in genomic analysis, providing essential insights into the organization of protein-coding genes, regulatory elements, and mobile genetic elements. The transcriptome, on the other hand, serves as a crucial link between an organism’s genome and its phenotypic expression (Velculescu et al., 1997). Currently, the most widely used method for transcriptomic analysis is RNA sequencing (RNA-seq), a high-throughput technology that enables comprehensive profiling of the transcriptome using next-generation sequencing platforms (Shendure, 2008).

The most widely recognized application of RNA-seq is the identification of differentially expressed genes in comparative experiments, such as those involving experimental and control conditions (Drewe et al., 2013). However, beyond this, RNA-seq technology has several other critical applications, including de novo transcriptome assembly (Cardoso- Silva et al., 2014), detection of genetic polymorphisms (Piskol et al., 2013), and the discovery of novel splicing variants. When sequencing and reconstructing the genomes of non-model organisms, transcriptome sequencing is often performed in parallel, as it significantly aids in genome annotation, prediction, and functional characterization of protein-coding genes. Nevertheless, due to the extensive genomic and morphological diversity within species, driven by structural variations, a single reference genome is insufficient to capture the complete gene repertoire of a species. To address this limitation, the concepts of pan-genome and pan-transcriptome have been introduced.

Reconstructing genomes and transcriptomes across a population enables the generation and analysis of pan-genomes and pan-transcriptomes in plants (Pronozin et al., 2021). The pan-genome concept encompasses sequences that are subject to structural variation and may be absent from the reference genome of a single representative of the species (Vernikos et al., 2015). Numerous studies have demonstrated that analyzing pan-genomes and pan-transcriptomes enhances the efficiency of research and increases the total number of predicted genes, compared to relying solely on the genome of a single representative (Jin et al., 2016). This approach improves the accuracy and completeness of the gene set under investigation.

Another area of bioinformatic analysis is the annotation of the genome and transcriptome. For protein-coding genes, an important part of their annotation is the identification of protein domains, a structural fragment of a protein that acts as an independent functional unit. It can form a unique structure or be part of multi-domain proteins, functioning both independently and in combination with other domains. For the functional identification of proteins, it is also significant to search already known genomes for orthologs, proteins that perform the same functions in different organisms.

Note also that more than 90 % of all transcripts are not translated into proteins (Carninci et al., 2005) and are noncoding sequences. Noncoding RNAs (ncRNAs) perform a number of important functions in plant genomes related to the regulation of gene expression and homeostasis of plant physiological parameters. One essential class of ncRNAs is long noncoding RNAs (lncRNAs) (Nazipova, 2021). The lncRNAs are a class of linear or circular RNA molecules 200 nucleotides or more in length that do not code proteins (Kim, Sung, 2012). The participation of lncRNAs has been revealed in the regulation of gene expression, formation of the structure of macromolecular complexes, interaction with proteins, and pathogenesis. To date, more than half a million lncRNA sequences have been identified for various organisms.

Data on gene expression levels obtained from transcriptomic experiments are widely used to reconstruct gene networks (Johnson, Krishnan, 2022). Gene networks, in turn, make it possible to model the dynamics of specific processes in an organism and predict its behavior under various conditions.

This paper presents the CropGene system for complex analysis of genomic, transcriptomic data, features of molecular evolution of agricultural plant genes. The system includes blocks of bioinformatic data analysis: analysis of gene variations, assembly of genomes and transcriptomes, annotation of genes and proteins.

## Materials and methods

**Fig. 1.**
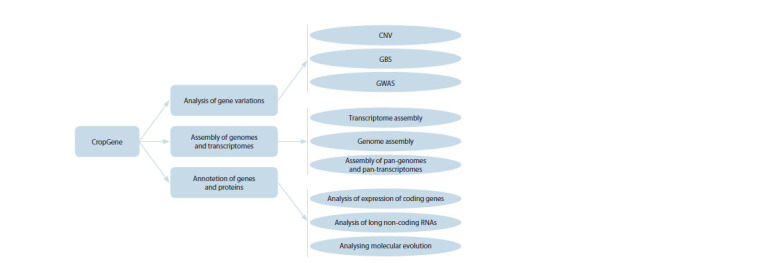
Diagram of the CropGene software package, with an indication of the main blocks of analysis (rounded rectangles in the center) and specific tasks to be solved (ovals on the right).

The CropGene software package includes the software packages shown in Figure 1.

The structure of the software package includes the following blocks for solving problems:

Module for the analysis of genome-wide associations.

This module implements the following analysis steps: • analysis of phenotyping data. The phenotyping data is processed using the R, pastecs, and psych packages (Grosjean et al., 2018), • processing of genotyping data. The step is aimed at processing genotyping data obtained by the microarray genotyping method and the GBS method. Processing includes the evaluation of raw data quality, mapping the reads to the reference genome using BWA-MEM (Li, 2013), and searching for genetic variants using vcftools (Danecek et al., 2011). The variants identified by the above genotyping methods are filtered by quality, minor allele frequency, heterozygosity, and the amount of missing data. This stage is performed by the bcftools instrument (Danecek et al., 2021). BEAGLE 5.2 is used to impute missing genotyping data (Browning et al., 2018), • genome-wide analysis of associations. At this stage, genome-wide association analysis is carried out. It is implemented in the R programming language using the functions of the GAPIT3 package (Wang, Zhang, 2021), • prioritization of genes at the identified loci. This module of genome-wide association analysis is aimed at identifying candidate genes associated with traits of interest. First of all, using the functions of the R “genetics” package, the boundaries of loci are determined, which include variants significantly associated with the phenotype. Further, based on published data on gene expression in the studied organism and on the resources of the Knetminer platform (Hassani‐Pak et al., 2021), the genes are prioritized among the detected loci.

Module for the CNV analysis. This module is aimed at solving the tasks of estimating and analyzing variations in the number of copies in the genome. It implements several stages of analysis:

• the sets of raw reads are filtered by quality and length using the fastp program (Chen et al., 2018). Then the filtered and processed reads are mapped to the reference genome of potato using the BWA program (Li, Durbin, 2009). Duplicates in the mapped reads are marked and deleted, after which the reads are sorted and indexed using SAMtools (Li et al., 2009), • the BAM files are used as input in CNVpytor (Suvakov et al., 2021). Copy number variations are detected on all chromosomes of the reference genome. The detected CNVs are filtered as follows: length greater than 1,000 bp, p-value <0.01, q0 < 50 %, pN < 50 %. The intansv R package is used to compare the identified CNVs with the genes of the reference genome (Jia et al., 2020), • for subsequent processing, the CNV list was presented in the form of a matrix in which the rows correspond to a specific genotype, and the columns correspond to the gene of the reference genome. Each element of the matrix is represented in three variants: +1 (potential duplication), –1 (potential deletion) and 0 (absence of a significant CNV). Next, principal component analysis (PCA) is performed using the Scikit-learn v1.1.2 package, which makes it possible to assess genetic diversity (Pedregosa et al., 2011).

Bioinformatic pipeline GBS-DP. This software module is aimed at analyzing the data obtained by the GBS method and consists of three main stages (Pronozin et al., 2023):

• data preprocessing includes checking the quality of raw FastQC readings, removing fast adapters (Chen et al., 2018), and building a reference genome index, • the search for polymorphisms consists of mapping preprocessed reads to the Bwa-Mem2 reference genome (Li, Durbin, 2009), sorting mapped SAMtools reads (Li et al., 2009), and searching for single-nucleotide polymorphisms Bcftools (Li, 2011), • the analysis of genetic diversity is divided into two data processing options: if the data obtained exceed the occupied memory capacity of 1 TB and if the data obtained do not exceed the occupied memory capacity of 1 TB. The appropriate option is selected automatically and is associated with an increased load on the computer’s RAM when working with big data. The R – SNPrelate package is used to analyze the main components filtered by SNPs (Zheng, 2013), and the SNPrelate package is used to build a phylogenetic tree.

Transcriptome reconstruction module. This module includes realisation of the following analysis stages:

• contig reconstruction from RNA-seq libraries. Several programs are implemented during this stage: Trinity (Grabherr et al., 2011), Trans-ABySS (Robertson et al., 2010), rnaSpades (Bushmanova et al., 2019), • aggregation of contig sets obtained during the previous stage and redundancy removal with the tr2aacds.pl tool from the EvidentialGene toolbox, • quality control of the resulting sequences; the BUSCO software (Simão et al., 2015) assesses transcriptome completeness; kallisto (Bray et al., 2016) shows percentage of initial RNA-seq libraries used in transcriptome reconstruction; rnaQUAST (Bushmanova et al., 2016) evaluates several metrics, including homology with genome sequence of reference organism or closely related organism in case the study is performed on non-model species.

Pangenome reconstruction and analysis module. This module implements the following analysis steps:

• reconstruction of each genome based on paired short reads using the MaSuRCA genome assembler (Zimin et al., 2013), • masking of mobile genetic elements using RepeatMasker and further de novo annotation of reconstructed masked genomes with further translation of open reading frames using the AUGUSTUS program (Stanke et al., 2004), • identification of orthologous groups in a set of amino acid sequences obtained on the basis of open reading frames using OrthoFinder (Emms, Kelly, 2019).

Gene expression evaluation module. The estimation of gene expression in this module can be performed either based on the reference genome or based on the de novo reconsructed transcriptome:

• to quantify the expression of reference genome genes, short read alignment to the genome sequence is performed using the Dart (Lin, Hsu, 2018) software. Next, based on genome annotation and known positions of genes, the number of reads mapped to each gene is estimated using the featureCounts (Liao et al., 2014) software, • to evaluate expression based on the previously reconstructed transcriptome, the kallisto software is used, which performs so-called pseudoalignment of reads to determine to which transcript they belong to; this allows for quantification of expression levels of these transcripts.

Bioinformatic pipeline ICAnnoLncRNA. This module, aimed at identifying and annotating lncRNAs, implements three stages of processing transcriptomic sequences (Pronozin, Afonnikov, 2023):

1) quality control. This stage includes two operations: the construction of an index file for the genomic sequence by the gmap program (Wu, Watanabe, 2005) and the training of the lncRNA recognition model by the LncFinder v1.1.4 program (Han et al., 2019). 2) lncRNA identification. This block consists of three stages: prediction of lncRNA candidates from the input set of transcripts using the LncFinder method; filtering of the obtained candidate sequences based on the identification of transmembrane segments in the OPC; alignment of filtered lncRNA candidate sequences to the reference genome, 3) annotation. The annotation includes the determination of lncRNA sequence types by alignment to proteincoding genes, identification of conserved lncRNAs, and analysis of the structural features of lncRNAs and their expression.

Module for analyzing protein evolution OrthoDOM. The module implements four key stages of protein sequence analysis:

1) the input data is validated and the presence of functional domains specified by the user for reference proteins is checked for, 2) the presence of key domains in the reference sequences is checked for, 3) the Orthofinder program runs for the studied proteomes, 4) the identified orthologs are checked for the presence of sets of specified domains in their sequence.

## Results and discussion

The modules of the CropGene software package have been used to solve various problems of bioinformatic analysis of genomes and transcriptomes of agricultural plants.

A software pipeline that detects CNVs based on genomewide data was previously used in the analysis of the structure of potato genomes of domestic varieties (Karetnikov et al., 2023). It allowed us to identify all the copy number variations in potato genomes and to conduct a comparative analysis of the number of copies of genes with South American potatoes. The analysis revealed that the frequency of CNV occurrence in four of the 48 known genes associated with tuber formation and photoperiod response differs between the genomes of Russian varieties adapted to long daylight hours in northern latitudes and local Andean cultivars adapted to short daylight hours.

**Fig. 2.**
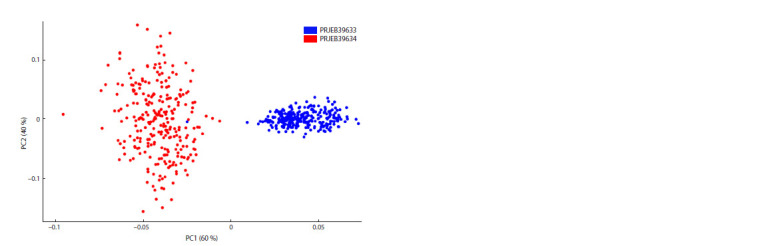
Visualization of the genetic diversity of 219 barley libraries using the PCA method. The first and second main components are directed along the X and Y axes, respectively.

**Fig. 3.**
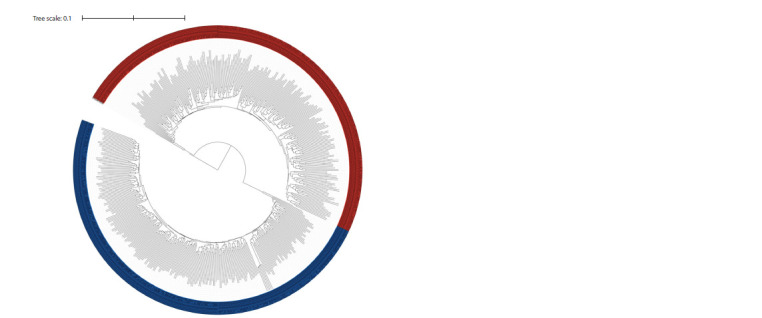
A dendrogram characterizing the genetic diversity of 219 barley libraries constructed by hierarchical clustering based on GBS data. The dendrogram is constructed on the basis of the found single nucleotide polymorphisms.

This work used GBS-DP to analyze 219 varieties of barley. 61,620 SNPs were identified. Based on the identified polymorphisms, clustering was performed using the principal component method (Fig. 2) and a dendrogram constructed using the hierarchical clustering method (Fig. 3).

The genome-wide association analysis module was used in the search for candidate genes of common winter wheat associated with pre-harvest sprouting and red grain color (Afonnikova et al., 2024). In addition to the discovery of genetic markers that explain up to 50 % of variability in grain lightness, red and blue color, the work has identified two candidate genes associated with the formation of grain color. The first gene, TraesCS1D02G319700, is located on chromosome 1D and participates in the synthesis of flavonols in the biosynthesis of flavonoids. The other gene,TraesCS7B02G482000, is localized on chromosome 7B and encodes phytoene synthase involved in one of the initial stages of carotenoid synthesis. The main candidate gene for the resistance to pre-harvest sprouting is the TraesCS6B02G147900 gene encoding the aleurone layer morphogenesis protein. Genetic markers were also identified that explain up to 25.3 % of the variability of pre-harvest sprouting traits – the germination index at the milk/hard dough stage of grain development. 

Based on the transcriptome analysis module, the transcriptome of four potato varieties of Solanum tuberosum group phureja (Bintier, Siverskij, Sudarynya, Evraziya) and wild-growing S. stoloniferum L. was constructed. Genes encoding proteins of the Nucleotide-binding site – Leucine rich repeats (NBS-LRR) family involved in the formation of the plant immune response were detected (Kochetov et al., 2021). It was found that the repertoires of these genes in the studied potato varieties and in wild nightshade differ significantly, which is consistent with the available data on the rapid evolution of these genes. Some of the NBS-LRR family genes observed in this work had not previously been detected in Solanaceae and potatoes in particular. These genes may become promising material for producing potato varieties that are more resistant to various pathogens and parasites.

**Fig. 4.**
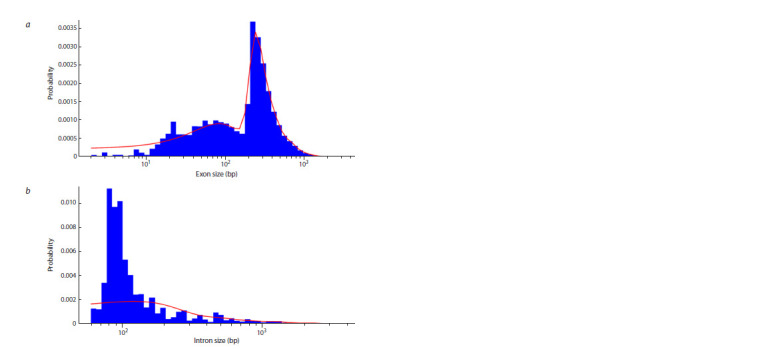
The ratio of the number of exons per lncRNA (a) and the distribution of the size of introns relative to lncRNA, respectively (b).

**Fig. 5.**
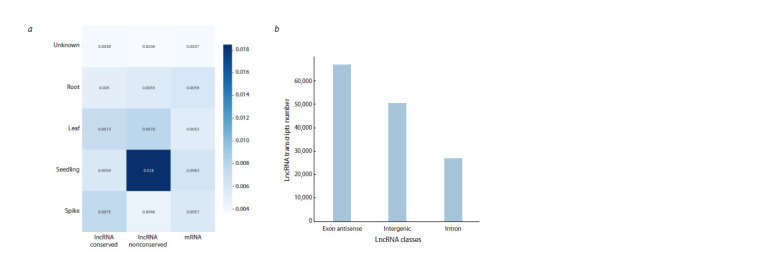
Specificity of mRNA expression in relation to various barley tissues, shown as a heat diagram. The X axis shows data for two classes of lncRNAs (conservative and non-conservative) and mRNAs. The correspondence of cell color and specificity value is shown by the scale to the right of the diagram (the higher the value in the cell, the more transcripts are specific to that tissue) (a), and the distribution of classes of barley lncRNAs (b).

The ICAnnoLncRNA pipeline was used to investigate 54 barley transcriptomes. 143,279 new lncRNAs were identified. Of these, 29,987 belong to the class of intronic lncRNAs, 48,369, to intergenic lncRNAs, 64,923, to antisense lncRNAs. Analysis of the lncRNA structure showed that the majority (60 %) contain only one exon. At the same time, the average exon length is 371 nucleotides, a small proportion of exons are up to 10 bp long, the vast majority are from 10 to 1,000 bp long, and their distribution has two characteristic peaks, one wide, with a maximum in the region of 100 bp, and the other narrow, in the region of 250–300 bp (Fig. 4). Tissue specificity analysis showed that the majority of lncRNAs are expressed in the tissues of barley sprouts (Fig. 5a). This is observed for both conservative and non-conservative lncRNAs. The same is true for mRNAs (Fig. 5a).

**Fig. 6.**
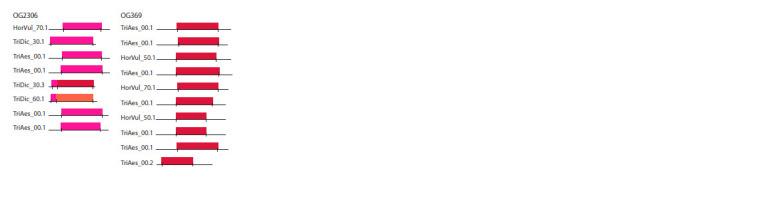
Domain structure of orthogroup sequences – 2306, 369, from left to right. The PLA2 beta domain is marked in red, PLA2 alpha is pink, and PLA2G12 is orange

The use of the OrthoDOM conveyor to detect phospholipase A2 family proteins in barley and wheat allowed us to confirm their presence in the genomes of these plants. During the study, two orthogroups were found. The domain structure (Fig. 6) of these groups shows marked similarity to the domain architecture of characteristic secreted A2 phospholipases (Larkin et al., 2019). The length of phospholipase A2 sequences in the orthogroups can be estimated as approximately 150 amino acids, with the PLA2 domain being the predominant part of the sequences, which corresponds to the known structure of secreted PLA2 forms.

## Conclusion

The developed CropGene software package includes the main blocks of programs necessary for the analysis of genomic and transcriptomic data of agricultural plants. These are blocks related to the assembly and analysis of the genome and transcriptome, including the formation of a pan-genome and a pan-transcriptome, analysis of GBS data, analysis of gene expression, recognition of long non-coding RNAs in plant transcriptomes, necessary for a comprehensive analysis of genomic, transcriptomic data, and features of the molecular evolution of agricultural plant genes. The use of these modules has made it possible to solve a number of important tasks in the analysis of genomic and transcriptomic data for crops such as potatoes, wheat, and barley.

## Conflict of interest

The authors declare no conflict of interest.
